# Primary central nervous system tumors survival in children in ten Colombian cities: a VIGICANCER report

**DOI:** 10.3389/fonc.2023.1326788

**Published:** 2024-02-27

**Authors:** Oscar Ramirez, Vivian Piedrahita, Jesus Ardila, Carlos Pardo, Edgar Cabrera-Bernal, John Lopera, Amaranto Suarez, Carlos Andrés Portilla, Carlos Narváez, Pamela Rodriguez, Ximena Castro, Ángel Castro, Diego Ivan Estupinan-Perico, Diana Valencia, María del Rosario Álvarez, Javier Enrique Fox, Luis Eduardo Bravo, Paula Aristizabal

**Affiliations:** ^1^ Unidad de Investigación, Fundación Pediatras Oncólogos y Hematólogos (POHEMA), Cali, Colombia; ^2^ Unidad de Oncología y Hematología Pediátrica, Clínica Imbanaco – Grupo Quirón Salud, Cali, Colombia; ^3^ Registro Poblacional de Cáncer de Cali – Departamento de Patología, Universidad del Valle, Cali, Colombia; ^4^ Escuela de Enfermería, Universidad del Valle, Cali, Colombia; ^5^ Unidad de Oncología y Hematología Pediátrica, Hospital de la Misericordia (HOMI) Fundación Hospital Pediátrico la Misericordia, Bogotá, Colombia; ^6^ Grupo de Oncología y Hematología Pediátrica Universidad Nacional de Colombia, Bogotá, Colombia; ^7^ Unidad de Oncología y Hematología Pediátrica, Instituto Nacional de Cancerología, Bogotá, Colombia; ^8^ Departamento de Pediatría, Universidad del Valle, Cali, Colombia; ^9^ Unidad de Oncología y Hematología Pediátrica, Fundación Valle del Lili, Cali, Colombia; ^10^ Departamento de Pediatría, Universidad de Cartagena, Unidad de Oncología y Hematología Pediátrica, Clínica Blas de Lezo, Cartagena, Colombia; ^11^ Unidad de Oncología y Hematología Pediátrica, Clínica Materno Infantil San Luis, Bucaramanga, Colombia; ^12^ Unidad de Oncología y Hematología Pediátrica: Instituto Médico de Alta Tecnología (IMAT) Oncomédica, Montería, Colombia; ^13^ Unidad de Oncología y Hematología Pediátrica, Hospital Infantil Los Ángeles, Pasto, Colombia; ^14^ Unidad de Oncología y Hematología Pediátrica, Fundación San Vicente de Paul, Medellín, Colombia; ^15^ Division of Pediatric Hematology/Oncology, Department of Pediatrics, University of California, San Diego, San Diego, CA, United States; ^16^ Pediatric Hematology/Oncology, Rady Children’s Hospital San Diego, San Diego, CA, United States; ^17^ Population Sciences, Disparities and Community Engagement, Moores Cancer Center, University of California, San Diego, San Diego, CA, United States; ^18^ Dissemination and Implementation Science Center, Altman Clinical and Translational Research Institute, University of California, San Diego, San Diego, CA, United States

**Keywords:** central nervous system neoplasms, pediatrics, treatment outcome, prognosis, epidemiology, Latin America, survival, children

## Abstract

**Purpose:**

Primary central nervous system (CNS) tumors are the second most common cancer in children and adolescents, leading to premature death and disability. Population-based survival estimates aid decision-making in cancer control, however data on survival for primary CNS tumors in Latin America is lacking. We describe survival rates for children with primary CNS tumors treated in ten Colombian cities.

**Methods:**

We analyzed data from children and adolescents newly diagnosed with cancer between 2012 and 2021, participating in the Childhood Cancer Clinical Outcomes Surveillance System (VIGICANCER) in ten cities in Colombia. VIGICANCER collects information on clinical outcomes from twenty-seven pediatric oncology units and conducts active follow-up every three months. VIGICANCER does not register craniopharyngiomas; we excluded intracranial germ cell tumors for this report. We used the Kaplan-Meier method to estimate the overall survival probability, stratified by sociodemographic variables, topography, WHO grading, receipt of radiation therapy, and type of surgical resection. We analyzed the prognostic capacity of variables using multivariate proportional Cox’s regression, stratified by city and year of diagnosis.

**Results:**

During the study period, VIGICANCER included 989 primary CNS tumors in 879 children and 110 adolescents. The cohort median age was 9 years; 53% of patients were males, and 8% were Afro-descendants. Most common tumors were supratentorial astrocytomas (47%), astrocytic tumors (35%), medulloblastomas (20%), ependymomas (11%), and mixed and unspecified gliomas (10%). Five-year overall survival of the entire cohort was 54% (95% CI, 51-58); for supratentorial gliomas, WHO grade I was 77%, II was 62%, III-IV was 27%, respectively, and for medulloblastoma was 61%. The adjusted hazard rate ratio for patients with WHO grade III and IV, for those with subtotal resection, for brainstem location, and for those not receiving radiation therapy was 7.4 (95% CI, 4.7–11.8), 6.4 (95% CI, 4.2–9.8), 2.8 (95% 2.1–3.8), 2.0 (95% CI, 1.3–2.8) and 2.3 (95% CI, 1.7–3.0), respectively.

**Conclusion:**

We found that half of Colombia’s children and adolescents with primary CNS tumors survive five years, compared to 70% to 80% in high-income countries. In addition to tumor biology and location, gross total resection was crucial for improved survival in this cohort. Systematic monitoring of survival and its determinants provides empirical data for guiding cancer control policies.

## Introduction

A wide range of morphologies characterizes primary central nervous system (CNS) tumors in humans, representing about 3% to 4% of all primary cancers (1, 2). Around 12% of all primary CNS tumors occur in children. (<15 years) (1, 2). CNS tumors are the second most common tumor occurring in children (2, 3), after leukemias, with an incidence (per million) displaying wide geographical variations, ranging from 1.7 in Yaoundé (Cameroon, 2004 to 2006) to 53.5 in Nebraska (USA, 1998 to 2012) (4). Differences in disease ascertainment and inclusion of non-malignant tumors partly explain the variations in incidence. In Latin America, the reported incidence of these tumors ranges from 17.9 in Ecuador (based on five population-based cancer registries (PBCR), 1993 to 2013) to 30.2 in Lima, Perú (2010 to 2012). The incidence rate in Colombia from 1992 to 2013 was 25.2, based on data from four PBCR cancer registries (4).

CNS tumors encompass tumors found in the brain, spinal cord, and meninges. Of these, brain tumors are the most frequent (3, 5, 6). In adults and children, tumor types mainly vary because children have a higher frequency of embryonal tumors, with medulloblastoma being the most frequent (7, 8). When planning treatment and evaluating its effectiveness in our era, it is crucial to consider the patient’s age, topography, histology, and molecular pathology (7, 9–11). Survival is the most important metric of therapeutic success (12), although life-altering disabilities in long-term survivors should also be considered. Most progress has been made in medulloblastoma, from 1960 to 2010, with five-year overall survival (OS) increasing from 23% to 73% (13).

The World Health Organization is leading the Global Initiative for Childhood Cancer, which aims to improve the survival of children with cancer (14). This initiative requires population-based survival estimates to tailor interventions and measure progress. Regular survival monitoring is crucial for evaluating advances in cancer care for children (15).

However, information about survival of children with CNS tumors in low-and middle-income countries (LMIC) is limited (13). In a recent systematic review of childhood CNS tumors population-based survival only five studies were conducted in LMIC, of which none was from Latin America (13). The Argentinian hospital-based pediatric oncology registry -ROHA- (16) reported (2012 to 2016) a three-year OS of CNS tumors of 64%, and a five-year OS of 52% for medulloblastoma (2005 to 2014). Our aim is to contribute to this knowledge gap by describing the survival of children with CNS tumors treated in 27 pediatric oncology units (POU) at ten Colombian cities.

## Methods

### Setting and study population

Colombia is located in South America's northwestern region and its population is 51 million inhabitants (17) with 12 million minors under 15 years old. Its 2022 per capita gross domestic product was 6664 US$, ranking 88th in the Human Development Index, with a score of 0.752 in 2021 (18). As of 2021, Colombia was the most unequal country in Latin America, with a GINI index of 0.542 (19), and a poverty rate after the pandemic peak of 39% (20).

VIGICANCER was established in Cali, the third largest city in Colombia, in 2009. VIGICANCER planning, methods, and implementation was previously published (21). VIGICANCER has expanded and currently encompasses 27 POU in ten Colombian cities, including approximately 55% of all childhood cancer cases predicted to occur annually in Colombia. This prediction is based on the estimated incidence of Cali’s PBCR (4). VIGICANCER has been approved by the ethics committee of each participating center and by the Universidad del Valle in Cali.

### Case definition

VIGICANCER includes individuals under 19 years with a new diagnosis of an invasive malignant neoplasm (5^th^ digit behavior code/3) as classified by the International Classification of Diseases for Oncology, third edition (ICD-O-3) (22). Tumoral behavior benign (/0) or uncertain (/1) are included only for CNS tumors. This benign or uncertain behavior of CNS tumors encompasses low-grade and optic pathway gliomas. The main ICD-O morphologic classification cases of benign or uncertain behavior included were: subependymal, giant cell astrocytoma (9384/1), pilocytic astrocytoma (9421/1), subependymoma (9383/1), myxopapillary ependymoma (9394/1), choroid plexus papilloma (9390/0), atypical choroid plexus papilloma (9390/1), angiocentric glioma (9431/1), choroid glioma of the third ventricle (9444/1), gangliocytoma (9492/0), ganglioglioma (9505/1), desmoplastic infantile astrocytoma and ganglioglioma (9412/1), dysembryoplastic neuroepithelial tumor (9413/0), central neurocytoma (9506/1), extraventricular neurocytoma (9506/1), cerebellar liponeurocytoma (9506/1), papillary glioneuronal tumor (9509/1), rosette-forming glioneuronal tumor of the fourth ventricle (9509/1), pineocytoma (9361/1), meningioma, not otherwise specified (NOS) (9530/0), atypical meningioma (9539/1), hemangiopericytoma, NOS (9150/1), and hemangioblastoma (9161/1). VIGICANCER also includes gliomas of the optic nerve (topographic code C72.3), whereas craniopharyngiomas are not included. As the basis for diagnosis, VIGICANCER uses the guide proposed by the International Agency for Cancer Research, where the most valid basis is microscopic (cytology or histology). However, a non-microscopic-based diagnosis is considered appropriate if a microscopic diagnosis is impossible. Non-microscopic diagnosis can also be based on specific tumoral markers (biochemical and/or immunologic) or by clinical investigation, which includes all diagnostic techniques (22). Clinical diagnosis only (without any diagnostic technique) is not considered sufficient for inclusion in VIGICANCER. Patients with a diagnosis by death certificate are accepted. To be included in VIGICANCER, the patient should also receive treatment in a POU in a participating city. The only exclusion criteria is for patients whose parents/legal guardians decline participation.

For this report, we included information on children and adolescents registered in VIGICANCER from January 1, 2012 to December 31, 2021, with tumors involving the following ICD-O-3 topography coding: meninges (C70.0 to C70.9), cerebrum (C71.0 to C71.4), ventricles (C71.5), cerebellum (C71.6), brain stem (C71.7), overlapping lesion of brain (C71.8), not otherwise specified topography of the brain (C71.9), spinal cord, cranial nerves, and other parts of CNS (C72.0 to C72.5), overlapping lesion of brain and CNS (C72.8), and not otherwise specified tumor in the nervous system (C72.9). The ICD-O-3 morphology codes included are shown in **Table 1**. WHO grading is used in VIGICANCER as reported in 2007 (23), which is also included in the ICD-O-3 (22).

**Table 1:** International Classification of Diseases for Oncology third edition (ICD-O-3) morphology codes for brain tumors, grouped by the International Classification of Childhood Cancer.

International Classification of Childhood Cancer			ICD-O-3 morphology codes
**III.a.**	**Ependymomas and choroid plexus tumors**		
	**III.a.1.**	Ependymomas	9383, 9391-9394, 9396
	**III.a.2.**	Choroid plexus tumor	9390
**III.b.**	**Astrocytomas**		9384, 9400-9411, 9420-9424, 9425, 9440-9442; 9380 (including optic glioma)
**III.c.**	**Intracranial and intraspinal embryonal tumors**		
	**III.c.1.**	Medulloblastomas	9470-9472, 9474-9478, 9480
	**III.c.2.**	Primitive neuroectodermal tumors	9473
	**III.c.3.**	Medulloepitheliomas	9501-9504
	**III.c.4.**	Atypical teratoid/rhabdoid tumors	9508
**III.d.**	**Other gliomas**		
	**III.d.1.**	Oligodendrogliomas	9450, 9451, 9460
	**III.d.2.**	Mixed and unspecified gliomas	9380 (excluding optic glioma)
	**III.d.3.**	Neuroepithelial glial tumors of uncertain origin	9381, 9430, 9431, 9444, 9445
**III.e.**	**Other specified intracranial and intraspinal neoplasms**		
	**III.e.1.**	Pituitary adenomas and carcinomas	8158, 8290, 8270-8281, 8300
	**III.e.2.**	Tumours of the sellar region (craniopharyngiomas)	9350-9352, 9432, 9582
	**III.e.3.**	Pineal parenchymal tumors	9360-9362, 9395
	**III.e.4.**	Neuronal and mixed neuronal-glial tumors	9412, 9413, 9492, 9493, 9505-9507, 9509
	**III.e.5.**	Meningiomas	9530-9539
**III.f.**	**Unspecified intracranial and intraspinal neoplasms**		8000-8005

### Variables

VIGICANCER actively collects the information from patients’ medical records, pathology reports, nurses administering chemotherapy, and social workers. Although some information is acquired directly from patients’ caregivers, in some POUs, access of VIGICANCER clinical monitors to patients’ caregivers has been restricted. Pediatric oncologists in each POU help in data quality checks and clarifying information when necessary. Centralized data quality checks are also performed.

We included demographic variables such as: age at diagnosis, sex, place of residence, afro-descendant ethnicity, and health insurance affiliation. We estimated the age of patients at diagnosis using the date of birth and divided it into five-year intervals. Participants who were diagnosed under the age of 15 were considered “children,” while those aged 15 to 18.9 were considered “adolescents.” VIGICANCER classifies sex and race/ethnicity (Afro-descendants vs. others) based on information from the medical record.VIGICANCER considers “place of residence” where the patient lived for at least six months before being diagnosed with cancer. We categorized the patients’ residential areas into those living in the capital city of a department with one or more POUs, those living in municipalities of departments with POU, those without POU, and patients residing abroad. We divided the cities based on the number of reported cases per year: large cities with ≥100 and small cities with <100 cases.

Colombia compulsory health insurance system is divided into contributory (for employees and self-employed) and subsidized categories (informal and low-income self-employed workers) (24, 25). Both insurance plans in Colombia cover 90% of the population. People not included in the above categories have health insurance through a government special plan for police, military, teachers, government employees, or private insurers. Around 4% of citizens are uninsured (26).

For CNS tumors we also included specific variables such as WHO grade (I to IV) (23), type of surgical procedure, amount of residual disease after surgery, receipt of adjuvant radiation therapy and/or chemotherapy. Only surgical procedures with diagnostic or oncological intention were registered (including biopsy-only procedures). Medulloblastoma was classified as “high” risk if the age at diagnosis was less than three years and/or gross total resection was not achieved with a residual tumor greater than 1.5 cm.

### Follow-up and outcomes

VIGICANCER conducts active follow-up every three months to monitor of the patient’s health status and gather information on the outcome variables. If VIGICANCER loses contact with a patient, passive surveillance is started using two different governmental social security information platforms to verify their vital status.

Four outcomes are measured: mortality, relapse, treatment abandonment and occurence of second neoplasms. Mortality is further classified into three categories: resulting from the tumor (caused by relapse or progressive disease), unrelated to the tumor occurring during cancer treatment, and unrelated to the tumor after cancer treatment completion. VIGICANCER uses the definition of treatment abandonment published by the International Society of Pediatric Oncology (27).

### Statistical analysis

We followed the group III categorization from the International Childhood Cancer Classification third version (ICCC-3) (28). In addition, we present information on supratentorial gliomas, which we have grouped according to WHO malignancy classification.

Crosstabulations were carried out between tumor groups and each variable. We used the maximum likelihood test or Fisher’s exact test to compare proportions, depending on the sample size.

For survival analyses, we estimated the time from the date of diagnosis to either the date of the event of interest or the last contact date for those without an event. The analysis cutoff date was August 31, 2023. We treated patients who abandoned cancer treatment whose vital status could not be verified as informed censorship and assigned an event at the treatment abandonment date. Patients lost to follow-up after cancer treatment were included in the analyses as censored observations if their vital status could not be determined through passive surveillance. Patients who were transferred to a non-VIGICANCER city during follow-up were also censored, however if their vital status was determined through passive surveillance, they were not censored in the analysis.

We used Kaplan-Meier to estimate the observed OS. We stratified survival by each variable and carried out the hypothesis testing of equal survival using the log-rank test.

We used conditional logistic regression to explore the potential association between partial or gross total resection and independent variables. Also, we evaluated whether if the association between brain stem tumors and Afro-descendant ethnicity was independent. We used as the grouping variable the city where the cases were registered. Additionally, we examined the independent prognostic capacity of the included variables by estimating adjusted hazard ratios (aHR) through multivariate proportional Cox’s hazards regression stratified by city and year of diagnosis. We evaluated the proportional hazards assumption for each model (29). We used STATA® v.17.0 and estimated 95% confidence intervals and considered a two tailed p value <0.05 as significant.

## Results

During the study period, VIGICANCER registered 7025 patients, including 989 primary CNS tumors, which comprised 879 children and 110 adolescents. Of the 989 CNS tumors registered, 985 had information available for follow-up. The median follow-up period for those still alive was 39 months, with a maximum of 114 months.

**Figure 1:**
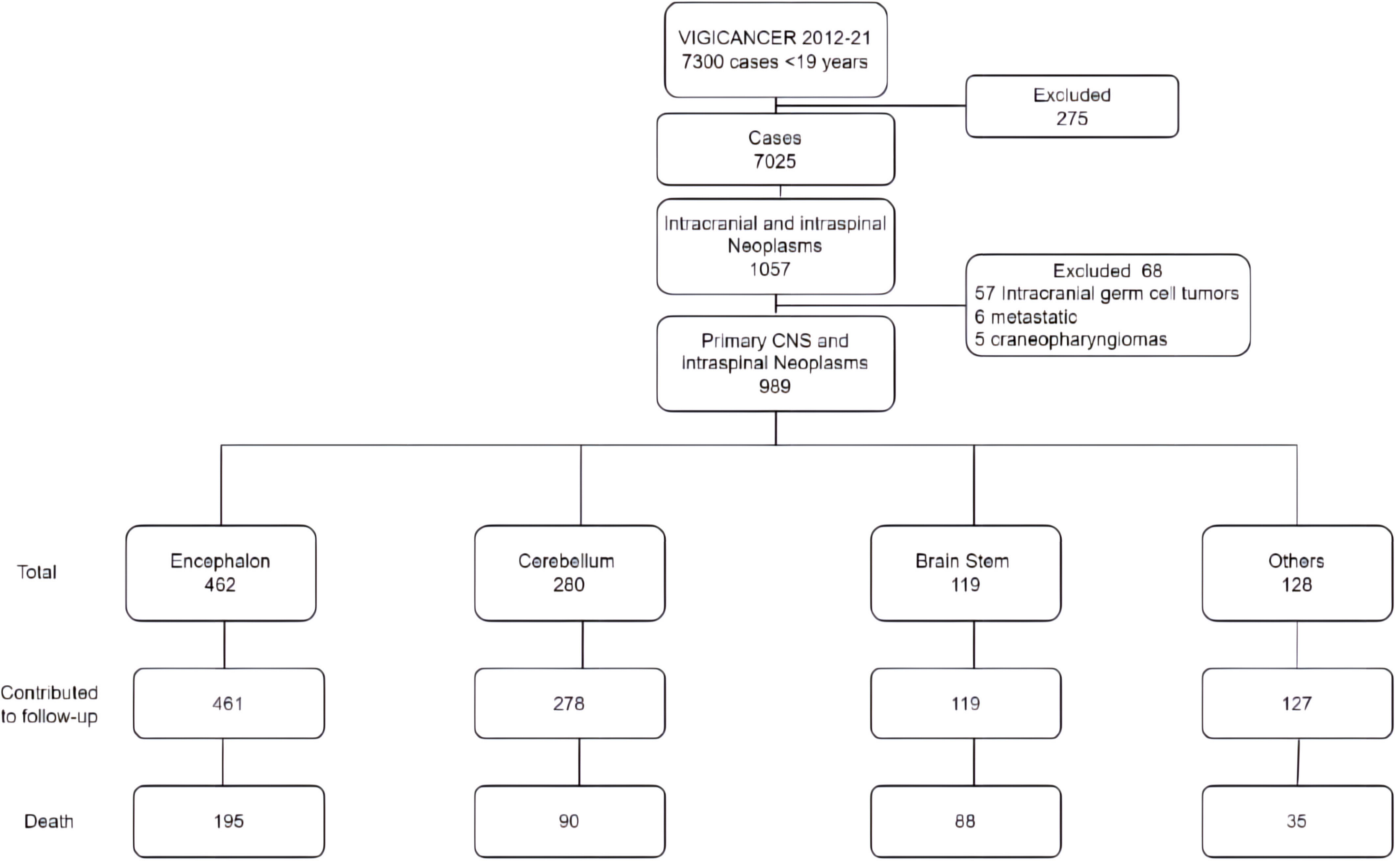
Flow chart of patients included in the analyses by topography.

Cohort median age was 9 years (IQR 4.8-12.6), 53% of patients were males, 8% Afro-descendants, 41% living in a city with POU, and 47% with subsidized health insurance (**Table 2**). In **Figure 1**, we show the flowchart of patients distributed by topography and **Table 3** ICCC grouping. The cerebrum (including the diencephalon) was the most commonly involved location (47%), followed by the cerebellum (29%), and brain stem tumors (12%). Cerebellar tumors were more frequent in boys (58% vs. 51% p=0.04) and brain stem tumors most frequent in girls (56% vs. 45%; p=0.02). Afro-descendants presented with more infratentorial tumors (cerebellar 38% and brain stem tumors 27%) compared to others. The frequency of Afro-descendant ethnicity in brain stem tumors was 17% and in the other category of 6% (p<0.01), with an aOR of 2.1 (95% CI, 1.2-3.7).

**Table 2:** Sociodemographic characteristics of the cohort.

Characteristics	N^a^	n	%
Age (in years)	989		
<1		38	4
1-4		219	22
5-9		322	33
10-14		300	30
15-18.9		110	11
Sex	989		
Boys		528	53
Girls		461	47
Afro-descendant	947		
Yes		72	8
No		875	92
Place of residence	984		
Capital city with POU^b^		400	41
Cities from a department with POU in the capital city		346	35
Cities from a department without POU		231	23
Other country		7	1
City size (cases/year)^c^	989		
≥ 100		764	77
<100		225	23
Health insurance type	972		
Contributory		443	46
Subsidized		456	47
Private insurance		23	2
Special insurance		39	4
Uninsured		11	1
International insurance		0	0
Gross total resection	799		
Yes		349	44
No		450	56

^a^N, Total number of cases; ^b^POU, Pediatric Oncology Unit; ^c^Number of cases registered per year.

**Table 3:** Distribution of primary CNS tumors in the cohort. The aggrupation is based on the International Classification of Childhood Cancer third version.

International Classification of Childhood Cancer			Total	
			n	%
**III.a.**	**Ependymomas and choroid plexus tumors**			
	**III.a.1.**	Ependymomas	106	11
	**III.a.2.**	Choroid plexus tumors	13	1
**III.b.**	**Astrocytomas**		357	36
**III.c.**	**Intracranial and intraspinal embryonal tumors**			
	**III.c.1.**	Medulloblastomas	201	20
	**III.c.2.**	Primitive neuroectodermal tumors	31	3
	**III.c.3.**	Medulloepithelioma	2	
	**III.c.4.**	Atypical teratoid/rhabdoid tumors	16	2
**III.d.**	**Other gliomas**			
	**III.d.1.**	Oligodendrogliomas	21	2
	**III.d.2.**	Mixed and unspecified gliomas	99	10
	**III.d.3.**	Neuroepithelial glial tumors of uncertain origin	20	2
**III.e.**	**Other specified intracranial and intraspinal neoplasms**			
	**III.e.1.**	Pituitary adenomas and carcinomas	2	0
	**III.e.2.**	Tumours of the sellar region (craniopharyngiomas)	0	0
	**III.e.3.**	Pineal parenchymal tumors	22	2
	**III.e.4.**	Neuronal and mixed neuronal-glial tumors	41	4
	**III.e.5.**	Meningiomas	9	1
**III.f.**	**Unspecified intracranial and intraspinal neoplasms**			
	---	Intraespinal neoplasms	26	3
	---	Unspecified intracranial	23	2
		**Total**	989	100

Overall, 91% of patients had a surgical procedure; patients with the lowest rates of surgical resections were those with brain stem tumors (64%; p<0.01), of which 87% were partial resection or biopsy-only procedures. Gross total resection was attained in 44% of cases included in the cohort, as shown in **Table 2**, and in 33% of patients under three years of age. Patients with residual tumor >1.5 cm were found in 56% of those with partial resections.

Three and five-year OS of the entire cohort were 57% (95% CI, 54-60) and 54% (95% CI, 51-58), respectively, as shown in **Table 4**. Children (<15 years) and adolescents (15-18.9 years) had similar 5-year OS (53% vs. 54%). Children under 24 months of age had a lower five-year-OS than older children (43% vs. 55%; P < 0.01). However, after adjusting for other variables, the aHR was 1.3 (95% CI, 0.8-2.0).

**Table 4:** Overall survival at 36 and 60 months of the most common CNS tumors by WHO grading.

**Tumor morphology**	**Scale**	**N^a^**	**n^b^**	**D^c^**	**Overall survival**			
					**36 months**		**60 months**	
					**%**	**(95% CI)**	**%**	**(95% CI)**
**All tumors**	**—**	989	985	408	57	(54 – 60)	54	(51 – 58)
**Ependymomas and choroid plexus tumors**	**Total**	119	118	44	62	(52 – 71)	57	(46 – 67)
	I	16	16	2	88	(59 – 97)	88	(59 – 97)
	II	59	59	20	65	(49 – 77)	61	(44 – 74)
	III	44	43	22	49	(32 – 64)	40	(23 – 56)
**Astrocytic tumors**	**Total**	358	355	157	55	(49 – 60)	52	(47 – 58)
	I	157	157	22	86	(79 – 91)	84	(77 – 90)
	II	64	62	25	60	(46 – 72)	57	(42 – 69)
	III	47	47	37	16	(7 – 28)	16	(7 – 28)
	IV	82	81	68	18	(10 – 27)	14	(7 – 23)
	Missing	8	8	5	—	—	—	**—**
**Other gliomas (including gliomas NOS)**	**Total**	117	117	67	39	(30 – 49)	29	(15 – 44)
	I-II	51	51	15	68	(52 – 80)	46	(11 – 76)
	III-IV^d^	40	40	35	11	(6 – 25)	—	—
	Missing	26	26	17	38	(20 – 56)	31	(13 – 51)
**Embryonal tumors**	IV	249	249	101	58	(51 – 64)	56	(49 – 63)
**Medulloblastomas**	IV	201	201	70	63	(55 – 70)	61	(53 – 68)
**Primitive neuroectodermal tumors**	IV	31	31	16	49	(30 – 65)	49	(30 – 65)
**Neuronal and mixed neuronal-glial**	I-III	41	41	10	75	(58 – 86)	75	(58 – 86)
**Pineal parenchymal tumors**	I-IV	24	24	8	65	(42 – 81)	59	(36 – 77)

^a^. N, Total number of cases; ^b^. n, number of cases which contributed to follow-up; ^c^. Deaths during the study period; ^d^. Twenty-four months survival estimates.

**Figure 2:**
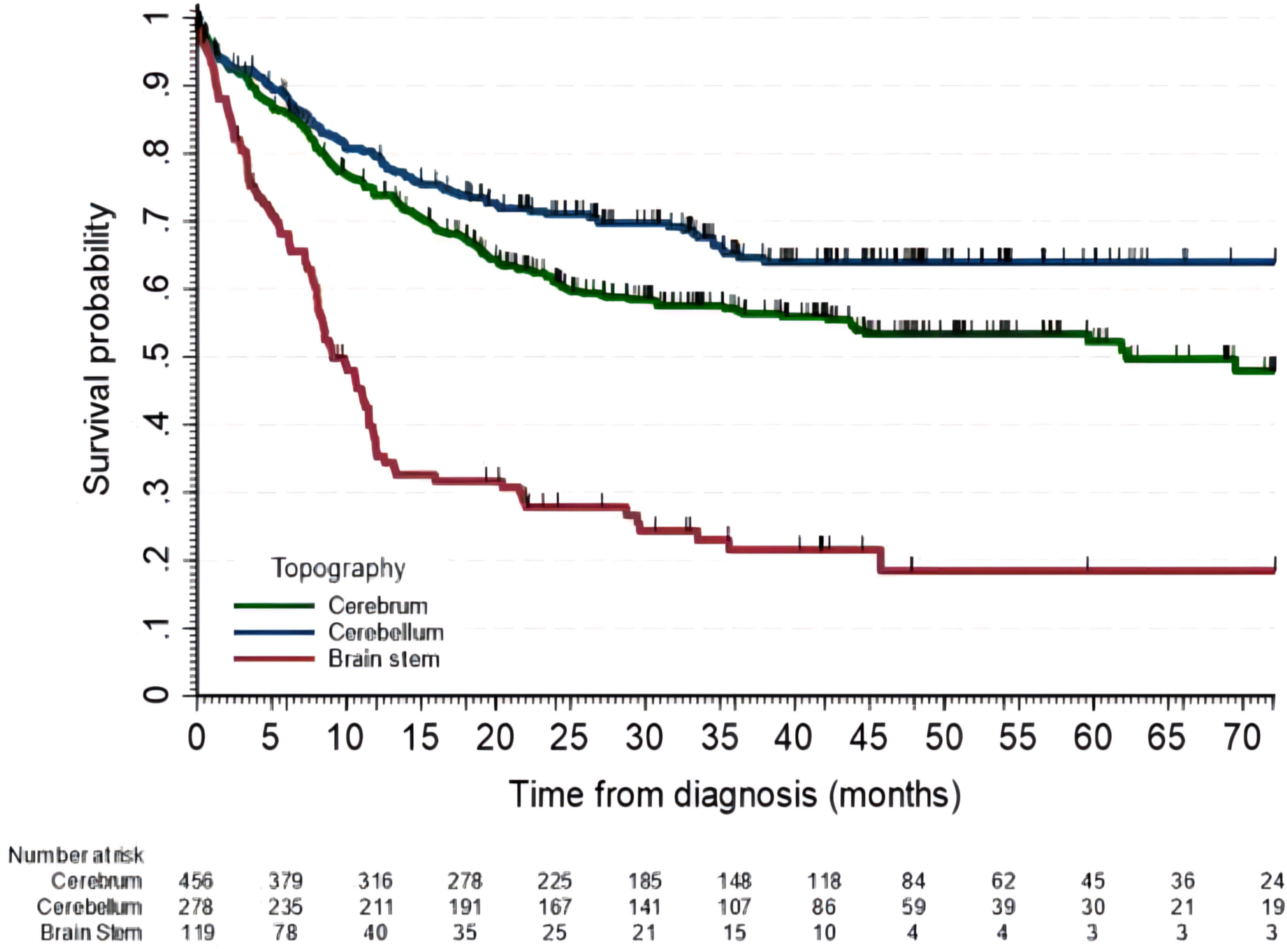
Overall survival of patients with cerebral, cerebellar and brain stem tumors. Five-year OS for cerebral tumors was 52% (95% CI, 47-58), for cerebellar 64% (95% CI, 58-70) and for brain stem tumors of 19% (95% CI, 11-28).

Patients who achieved gross total resection had a higher 5-year OS of 76% (95% CI, 70 - 80) than those with a partial resection (48%; 95% CI, 41-54) and than the group with biopsy only [30% (95% CI, 21-39)]. Patients who did not achieve gross total resection had a higher mortality risk with an aHR of 2.8 (95% CI, 2.1-3.8) while those who had biopsy only had an aHR of 4.8 (95% CI, 3.3-7.0). Cases registered as not receiving radiation therapy were independently associated with higher mortality risk with an aHR of 2.3 (95% CI, 1.7-3.0). WHO grading was also associated with increased risk of death with aHR for grade II of 2.7 (95% CI, 1.7-4.4), for grade III of 7.4 (95% CI, 4.7-11.8), and grade IV of 6.4 (95% CI, 4.2-9.8); as shown in **Table 5**. We did not observe significant differences by sex, ethnicity, place of residence, health insurance type, year of diagnosis, or receipt of chemotherapy. **Figure 2** displays survival curves for cerebral, cerebellar, and brainstem tumors, with the worst survival (aHR of 2.1, 95% CI: 1.4-3.0).

**Table 5:** Multivariate Cox's proportional hazards regression models^a^.

Variables		Model 1		Model 2	
		HR^b^	(95% CI)	HR	(95% CI)
**Gross total resection**	Total	**Ref.**		**Ref.**	
	Subtotal	2.7	(2.0 – 3.7)	2.8	(2.1 – 3.8)
	Biopsy-only	5.1	(3.5 – 7.5)	4.8	(3.3 – 7.0)
	Missing	6.3	(2.0 – 19.3)	5.4	(1.9 – 15.8)
**Brainstem tumors vs. others**		2.1	(1.4 – 3.0)	2.0	(1.3 – 2.8)
**WHO grading**	I	**Ref.**		**Ref.**	
	II	2.8	(1.7 – 4.6)	2.7	(1.7 – 4.4)
	III	7.7	(4.8 – 12.2)	7.4	(4.7 – 11.8)
	IV	6.5	(4.2 – 10.1)	6.4	(4.2 – 9.8)
	Missing	2.6	(1.2 – 5.7)	2.5	(1.1 – 5.6)
**Receipt of adjuvant radiation therapy**	Yes	**Ref.**		**Ref.**	
	No	2.1	(1.5 – 2.9)	2.3	(1.7 – 3.0)
	Missing	0.3	(0.0 – 3.2)	0.8	(0.2 – 3.0)
**Age <2 vs. ≥2 years old**		1.3	(0.8 – 2.0)		
**Boys vs. girls**		1.1	(0.8 – 1.4)		
**Afro-descendant**	No	**Ref.**			
	Yes	0.6	(0.4 – 1.0)		
	Missing	0.7	(0.1 – 6.1)		
**Other vs. capital with pediatric oncology unit**		1.0	(0.8 – 1.3)		
**Uninsured vs. insured**		0.7	(0.2 – 2.2)		
**Receipt of adjuvant chemotherapy**	Yes	**Ref.**			
	No	1.1	(0.8 – 1.5)		
	Missing	3.0	(0.4 – 22.7)		

^a^Regression analyses performed over the patients that had any kind of surgical intervention 814 cases. Model 1, saturated model with 780 cases model; Model 2, more parsimonious model with 789 cases; ^b^HR, hazard ratio.

### Ependymomas and choroid plexus tumor

During the study period, 106 ependymomas and 13 choroid plexus tumors were registered, representing 12% of all CNS tumors. This group of tumors was most frequently diagnosed in children under three years of age vs. older age [21% vs. 11%; aOR 2.3 (95% CI, 1.5-3.8)]. We did not find differences between sex, ethnicity, insurance type, place of residence, or year of diagnosis.

Ependymomas WHO grading II were the most frequent at 49%, followed by grade III at 40%, and grade I at 11%. Out of the 110 cases for which information was available, 109 received a surgical intervention. In 47% of the cases, resection was considered partial, and in 7%, only a biopsy was performed.

The 5-year OS for patients with ependymomas and choroid plexus tumors was 57% (95% CI, 46-67). **Table 4** shows survival according to the WHO’s scale. Patients under the age of 11 years had a lower 5-year OS rate of 47% (95% CI, 34-59) compared to older patients with a rate of 79% (95% CI, 55-91).

In the group that underwent surgical intervention, those with gross total resection had 5-year OS of 73% (95% CI, 54-85), which was higher than those with partial resection or biopsy only intervention [48% (95% CI, 33-61)].

In the multivariate analysis, patients under the age of 11 years [aHR of 4.4 (95% CI, 1.2-15.7)], those with subtotal resection [aHR of 2.9 (95% CI, 1.1-7.1)], and those with infratentorial location [aHR of 3.5 (95% CI, 1.1-10.8)], were independently associated with an increased rate of death.

### Astrocytoma, oligodendrogliomas, mixed and unspecified gliomas, and neuroepithelial glial tumors of uncertain origin

Astrocytic tumors represented 36% of all CNS tumors, and were classified as WHO grade I in 44%, grade II in 18%, grade III in 13%, grade IV in 23%; and data missing in 2% of cases. Supratentorial astrocytomas represented 47% of all CNS tumors. Two-thirds of astrocytic tumors occurred among children 5 to 14 years of age and were slightly more frequent in boys (53%) than in girls. Total resection was achieved in 36% of cases.

Two percent of CNS tumors were oligodendrogliomas, 10% mixed and unspecified gliomas, and 2% neuroepithelial glial tumors of uncertain origin (**Table 3**). Oligodendrogliomas were most commonly diagnosed in children over ten years old (71%) and had a similar sex distribution to other patients in the cohort. Additionally, 76% of these tumors were supratentorial, and 60% were classified as WHO grade II. Mixed and unspecified gliomas were most frequent between 5 to 9 years of age (42%). Sixty percent ocurred in girls, which was a higher frequency than for other CNS tumors (40%; p<0.01), with similar distribution between supra and infratentorial locations, and the majority were grade I (63%). Neuroepithelial glial tumors of uncertain origin were found in 90% of patients over five years old, with no sex predominance. Overall, 80% of tumors were supratentorial and 72% were WHO grade I.

**Figure 3:**
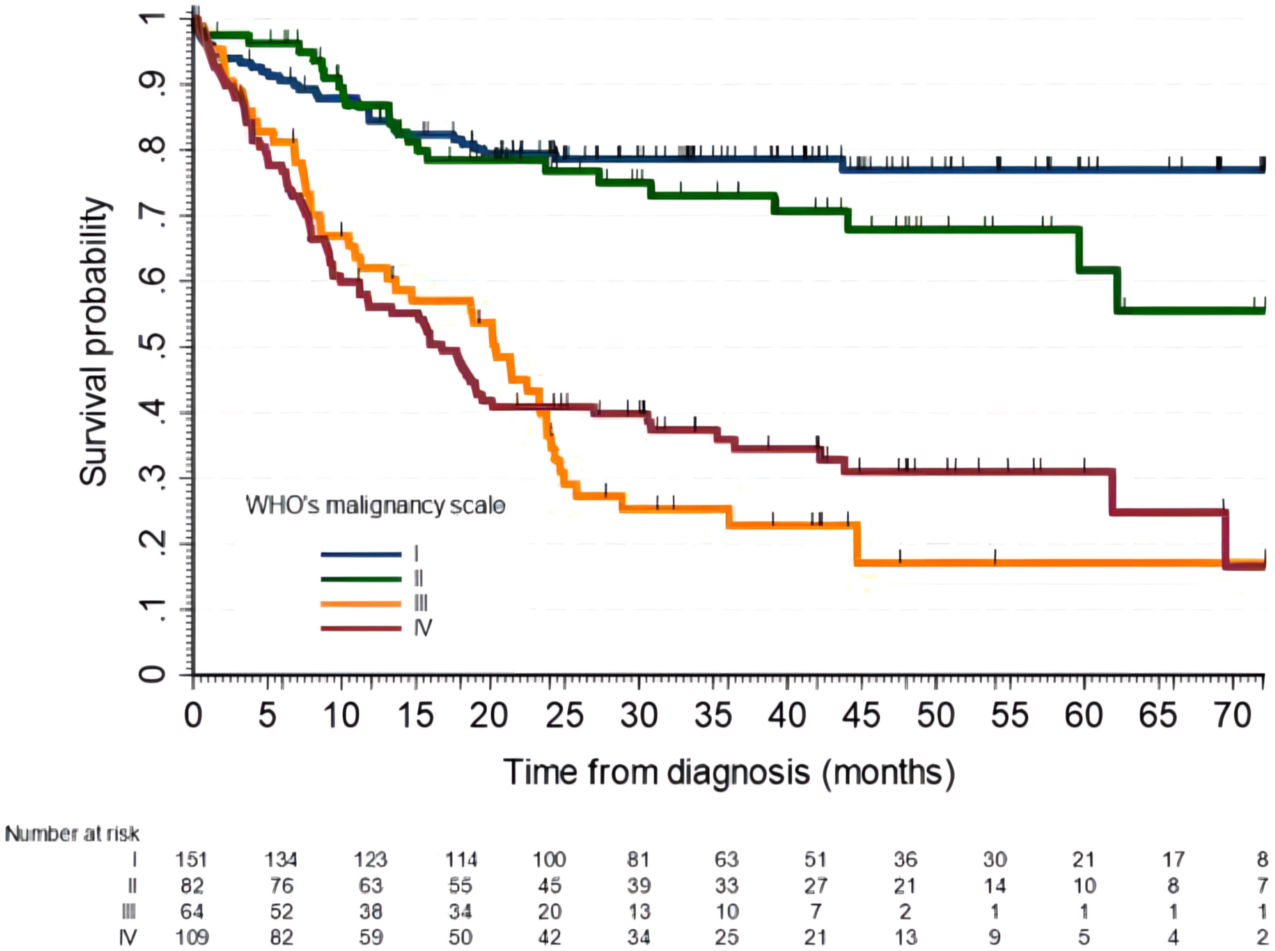
Overall survival of patients with supratentorial gliomas by the WHO malignancy scale. Five-year OS for grade I was 77% (95% CI, 69-83), for grade II was 62% (95% CI, 44-75), grade III 17% (95% CI, 7-32) and grade IV 31% (95% CI, 22-41).

Five-year OS for astrocytic tumors and other gliomas is detailed in **Table 4**, and OS survival curves for supratentorial glioma by WHO grading are shown in **Figure 3**.

For supratentorial gliomas, the fact of not attaining gross total resection was independently associated with a higher risk of death with an aHR of 3.7 (95% CI, 2.3-5.7).

### Intracranial and intraspinal embryonal tumors

Embryonal tumors comprised 25% of all CNS tumors. The majority of these were medulloblastomas at 81%, followed by primitive neuroectodermal tumors at 12%, atypical teratoid/rhabdoid tumors at 6%, and medulloepitheliomas at only 1%.

We found that one-third of all embryonal tumors were diagnosed in children under five years of age. Among this age group, the most common embryonal tumors were atypical teratoid/rhabdoid tumors (81%), followed by primitive neuroectodermal tumors (47%) and medulloblastomas (25%). Most of these cases occurred in boys (61%), and 10% were found in individuals of African descent.

Median age in children with medulloblastoma was 8 years (IQR, 5-12), 63% were boys and 10% were afro-descendants. Classic medulloblastoma was the most frequent histology (78%), followed by desmoplastic (16%), large cell (4%), medullomyoblastoma (1%), and not otherwise specified (1%). Medulloblastomas were totally resected in 58% of the patients. Children under three years of age had a higher risk of not achieving gross total resection, with an aOR of 3.1 (95%, 1.0-9.2). We did not observe an association between resection and sex, ethnicity, insurance type, city size, or year of diagnosis. Among those who did not undergo gross total resection, 13% underwent biopsy only. A residual tumor greater than 1.5 cm was found in 47% of cases. Radiation therapy and chemotherapy were administered as adjuvant therapy in 76% of patients with medulloblastoma, with radiation therapy given in 59% of high-risk patients and in 29% of cases under three years old.

**Table 3** shows the five-year OS for embryonal CNS tumors. Out of the 16 individuals diagnosed with atypical teratoid/rhabdoid tumors, only one has survived after a follow-up of 23 months. Meanwhile, the two patients who had medulloepithelioma have survived for 51 and 108 months since their diagnosis.

Children under the age of three who had medulloblastoma had a lower 5-year OS of 32% (95% CI, 11-55) compared to older children with OS of 65% (95% CI, 56-72) and an increased risk of death with an aHR of 2.6 (95% CI, 1.2-5.7). Those between the ages of 1 and 4.9 had a 5-year OS of 49% (95% CI, 43-63). Patients with contributive health insurance had a 5-year OS of 67% (95% CI, 55-77), while those with subsidized insurance had an OS of 57% (95% CI, 45-67) and an increased risk of death with an aHR of 2.1 (95% CI, 1.1-4.1).

**Figure 4:**
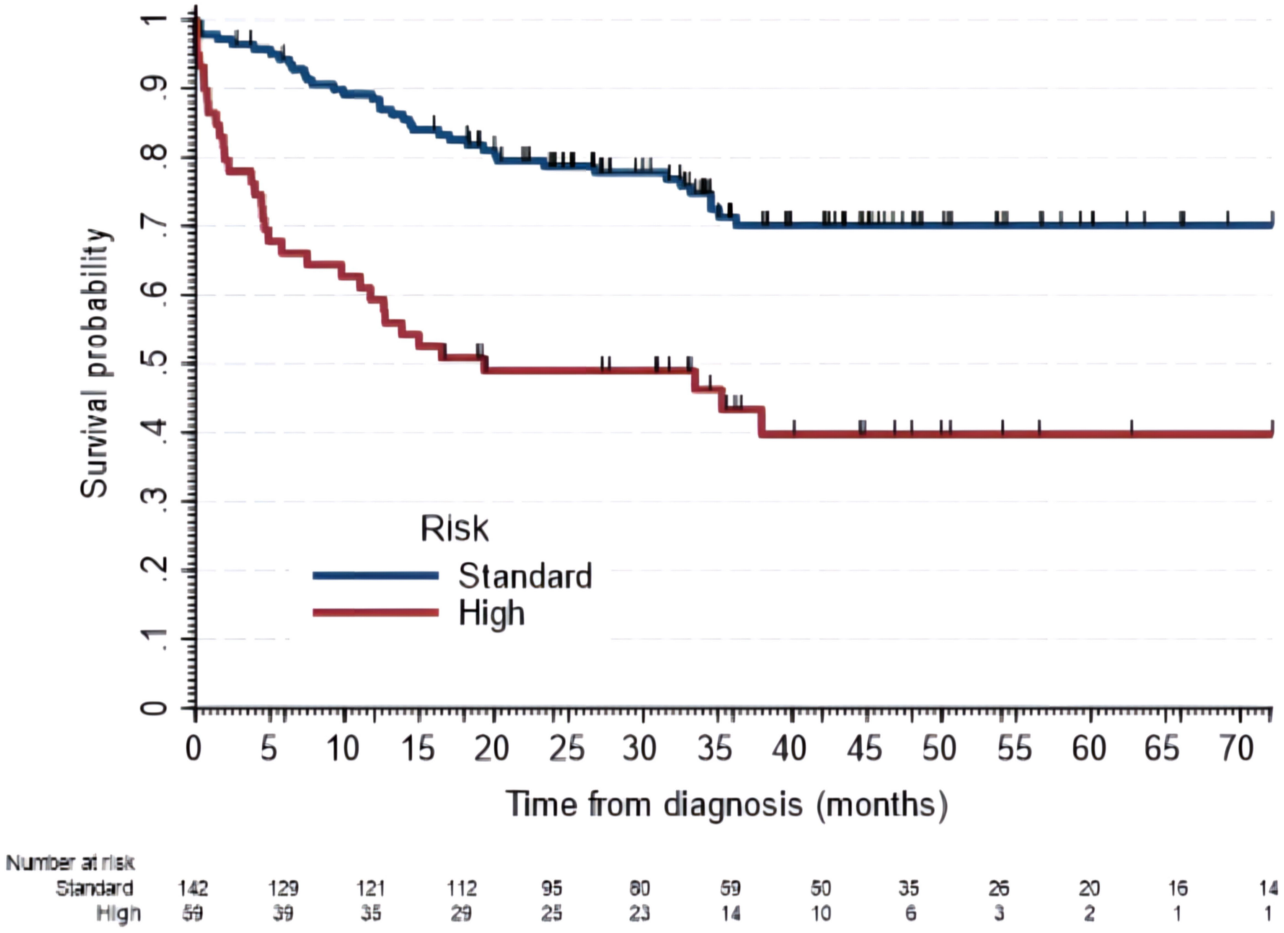
Overall survival of patients with medulloblastomas by risk classification. Five-year OS for the standard risk was 70% (95% CI, 60-78) and for high risk was 40% (95% CI, 26-54).

Children with classic and desmoplastic types had similar 5-year OS (63% vs. 61%). Eight patients had large cell medulloblastomas, of which only three were alive with a maximum follow-up of 48 months. Five-year OS for children under three years of age was 32% (95% CI, 11-55), lower than the older group [65% (95% CI, 56-72)]. Similarly, for the high-risk group, OS was 40% (95% CI, 26-53) whereas for the standard group it was 70% (95% CI, 61-78) as displayed in **Figure 4**. Those without gross total resection showed a 5-year OS of 54% (95% CI, 41–65), which was lower than those with total resection of 70% (95% CI, 60-79). Children under fiver years of age and without gross total resection had a 5-year OS of 27% (95% CI, 8-49), compared to those with gross total resection who had an OS of 64% (95% CI, 43-79). High-risk medulloblastomas showed an increased mortality risk with aHRs of 3.9 (95% CI, 2.3-6.8), in children with subsidize insurance of 2.0 (95% CI, 1.1-3.7) and in those without insurance of 3.5 (95% CI, 1.0-12.0).

### Other tumors

Three percent of CNS tumors were spinal cord and cranial nerve tumors, 2.5% were pineal tumors, 1% (13 cases) were optic gliomas, and less than 1% (9 cases) were meningiomas. Pineal tumors showed the lowest 5-year OS in this group at 59% (95% CI, 36-77). All optic gliomas were alive at the end of the study period.

## Discussion

In this national multicenter prospective cohort in a Latin American middle-income country, we found that children and adolescents with primary malignant and non-malignant CNS tumors had 54% five-year survival after diagnosis. This survival estimate is lower than estimates reported in high-income countries, which range between 70% and 80% (12), with the Central Brain Tumor Registry of the United States (CBTRUS, cohort 2014 to 2018) estimated at 83% (30). EUROCARE-6 survival for CNS tumors has been reported at approximately 60%, with significant heterogeneity across countries (15).

The CONCORD program for Colombia (2000 to 2014) reported a similar survival of approximately 47% (36% to 58%) based on data from four PBCRs(12). The similarities between VIGICANCER and CONCORD survival estimates suggest that VIGICANCER can approximate population-based survival probabilities. It also indicates that more progress needs to be made in childhood primary CNS tumor survival in our country. According to CONCORD-3 estimates (12), the results are comparable to Ecuador (48%) and Mexico (37%) but lower than Argentina (63%). However, the ROHA reports a five-year OS for CNS tumors of 56%, closer to our estimate (16).

We observed an almost three times increase in risk of death in children not achieving gross total resection. The prognostic role of gross total resection in children is not entirely settled (31–34). Uncertainty about its role increases with the progress into molecular classification and directed therapy (32). The Cross-Border Neuro-Oncology Program (San Diego, California- Tijuana, Mexico) (35) showed an increasing survival trend associated with attaining a higher proportion of patients with gross total resection. In our cohort, we did not find an association between the patient’s age and gross total resection.

### Ependymomas and choroid plexus tumor

We found that the five-year OS for ependymomas and choroid plexus tumors was 57%, lower than the one reported by EUROCARE-5 of 70% (36), and the one cited by CBTRUS of 89% (30). However, our survival estimates are similar to the ones described by ROHA of 61% (16). Nevertheless, patients with WHO grade I malignancy in our group had a five-year OS of 92%, which is congruent with the 97% reported in EUROCARE-5 (36).

### Astrocytic tumors

We found a 52% five-year OS for astrocytic tumors, which is 28% lower than the reported by EUROCARE-5 of 80% (36). The main survival gap in these tumors (EUROCARE-5 vs. VIGICANCER) was for grade I (11% lower in VIGICANCER) and for grade II (18% lower in VIGICANCER), which are the most curable astrocytic tumors (15). Survival for all supratentorial gliomas was 49%, with OS for high-grade gliomas being approximately half of the one reported by CBTRUS (15% vs. 33%) (30). Patients with astrocytic tumors have a three to four times higher risk of death if they do not achieve a complete gross total resection, regardless of other factors.

### Medulloblastoma

For medulloblastoma, the five-year OS was 61%, which is lower than the current estimate of 74% (72-75%) for under 19 years in the United States (2014-2018) (30), and close to ROHA’s estimate of 52% (16). We did not find higher survival estimates in children with desmoplastic medulloblastoma, contrasting with published literature. In our cohort, survival outcomes in children with medulloblastoma were significantly influenced by age, with those under three years old having only a 32% five-year OS. The group between one to four years old showed significantly lower OS (49%) compared to the observed survival probability in the United States (30). Children under age five with medulloblastoma who did not attain gross total resection had a five-year OS of only 27%, compared to 64% for those with gross total resection. This is consistent with the survival (64%) reported in the United States for children in the same age group. The difference in survival between our estimates and those of higher-income countries could be, at least, partially explained by the ability to achieve a gross total resection (35).

High-risk medulloblastoma classification includes two strong independent prognostic factors: age and achieving a gross total resection. Patients classified as high-risk had nearly four times the risk of death compared to those classified as standard risk. We also observed a higher risk of death for those without health insurance, underscoring the importance of a universalized health system to improve clinical outcomes (37). Since 2018, we included molecularly defined histopathologies for medulloblastoma in VIGICANCER. Nevertheless, currently the routine application of molecular classification is seldom used in Colombia and, therefore, we do not have enough cases for analysis. We expect that the completeness of this variable will increase in future years.

### Other embryonal tumors

Primitive neuroectodermal tumor survival was 49% which is like the figure reported by EUROCARE-5 of 41% (36), but lower than the reported by the CBTRUS of 64% (30). Atypical teratoid/rhabdoid tumor has a dismal prognosis with only 1 patient surviving in our cohort, while survival in EUROCARE (36) and CBTRUS were 23% and 33%, respectively (30).

In conclusion, our survival estimates are congruent with those reported in the German 1990–1999 cohort; with OS for astrocytic grade I-II at 82%, grade III-IV at 24%, medulloblastomas at 53%, and ependimomas at 57% (6). Brainstem tumors had a five-year OS of 19%, which is close to the one reported by ROHA of 22% (16), but much lower compared to reports by CBRTUS of 58% (30).

Our findings support the urgent need to improve treatment for childhood CNS tumors in Colombia. Despite universal health coverage and granted access to childhood cancer treatment, delays in diagnosing CNS tumors persist due to inadequate primary care services and inefficent referral pathways due to several health system organizational barriers. Therefore, strengthening primary care services to quickly detect childhood brain tumors and a straightforward referral to a higher complexity healthcare facility can improve clinical outcomes (38, 39). It is also crucial to enhance diagnostic capacities (number of neuropathologists, centralizing the diagnosis, standardizing reports, including molecular diagnosis), neurosurgical (increasing the proportion of gross total resections and decreasing sequelae), and clinical supportive care capacities, social support services, as well as timely access to radiation therapy. One way going forward is to centralize these patients in specialized centers (38, 40–42). However, in Colombia, this option is currently hindered by the fragmented healthcare system and the dependence of clinical services on unstable insurance contracts.

### Study limitations

Our study found that the distribution of tumors based on morphology, topography, and demographics was similar to other reports. However, making direct comparisons with published literature has several challenges. Our study did not include craniopharyngiomas and intracranial germ cell tumors, and we looked at both malignant and non-malignant primary CNS tumors. Our findings were based on pathology reports from treatment centers and did not undergo centralized diagnostic validation. This report is based on 27 POUs, and although those with the highest number of cases diagnosed per year in Colombia are in VIGICANCER, not all POUs are included. Additionally, the population representation of cases decreases as we analyze data from early periods, since the addition of cities to VIGICANCER has been a gradual process over the last decade. Therefore, it is worth noting that our study was not absolutely population-based and cannot estimate the incidence rates of the tumors we examined. In addition, there may be some uncertainty regarding the accuracy of our survival estimates compared to the population estimates. However, as stated previously, our survival estimates fall within the CONCORD (13) population-based survival ranges, indicating that if there was a selection bias, it did not substantially affect our assessments. We consider that VIGICANCER’s underestimation of the number of CNS tumors affected mainly adolescents, as its primary data source are POUs. Some adolescents with cancer in Colombia continue to receive treatment from adult oncologists. In Colombia, we have great uncertainty about how many patients with brain tumors are not diagnosed in the country and are contributing to the incidence gap. Statistical modeling has estimated this incidence gap to be 29% for upper-middle-income countries (38). Nevertheless, VIGICANCER’s comprehensive geography coverage, high number of participating centers, and low cohort attrition are strengths of this report. We estimate that currently, VIGICANCER covers about 55% of all childhood cancers expected to occur in Colombia.

In summary, this report presents the survival estimates and prognostic factors of primary CNS tumors in Colombian children and adolescents. Overall, age under two years, extent of resection, and WHO’s grade were independent prognostic factors. We used data from VIGICANCER, a surveillance system for the systematic monitoring of clinical outcomes of pediatric cancer patients in Colombia. This system provides empirical data that can be used to inform cancer control policies.

## Data availability statement

The raw data supporting the conclusions of this article will be made available by the authors, without undue reservation.

## Author contributions

OR: Conceptualization, Data curation, Formal analysis, Funding acquisition, Investigation, Methodology, Project administration, Resources, Software, Supervision, Validation, Visualization, Writing – original draft, Writing – review & editing. VP: Data curation, Investigation, Methodology, Project administration, Validation, Writing – review & editing. JA: Funding acquisition, Project administration, Writing – review & editing. CP: Writing – review & editing. EC-B: Writing – review & editing. JL: Writing – review & editing. AS: Writing – review & editing. CP: Funding acquisition, Resources, Writing – review & editing. CN: Writing – review & editing. PR: Writing – review & editing. XC: Writing – review & editing. AC: Writing – review & editing. DE-P: Writing – review & editing. DV: Writing – review & editing. MÁ: Writing – review & editing. JF: Writing – review & editing. PA: Conceptualization, Methodology, Writing – review & editing. LB: Conceptualization, Investigation, Methodology, Software, Writing – review & editing.

## Group member of VIGICANCER working group

The VIGICANCER working group includes the following co-investigators listed below alphabetically by surname and city. **Bogotá**: Ingrid Aristizabal, Gisella Barros, Edgar Cabrera, Carolina Casas, Angélica Castillo Plata, Agustín Contreras, Mauricio Chaparro, Marcela Estupiñán, Jessica Flechas, Natalia González, Oscar González, Carolina Guzmán, Jorge Hernández, Ana María Infante, Jimmy Lagos, Adriana Linares, Edgar Lopera, Leila Martínez, Mauricio Mesa, Carlos Pardo, Jaime José Pérez Niño, Martha Piña, Amaranto Suárez. **Bucaramanga:** Diego Iván Estupiñán Perico, David José Garay, María del Pilar Obregón Martínez, María Alexandra Pérez Sotelo, Julio Andrés Ropero, Diana Lorena Valencia. Cali: Jesús Ardila Novoa, Liliana Barragán, María Ximena Castro García, Ruth Castro, Alexis Franco, Viviana Lotero Díaz, Carlos Eduardo Narváez, Remberto Osuna, Carlos Andrés Portilla, Diana Soraya Rendón Ceballos, Pamela Andrea Rodríguez, Luis Hernán Romero, Luz Angela Urcuqui, Diego Medina Valencia. **Cartagena:** Ángel Castro Dajer, Ayslin González Cabarcas, Heidy Marsiglia Armella, Zoraya Paternina. **Ibagué**: Francy Helena Ortiz, Daniel Ozaeta, Dra. Patricia Montenegro. **Manizales:** Dra. Bibiana Villa Rosales. **Medellín:** Javier Enrique Fox Quintana, María Rosmira Pérez, Lina Quiroz, Carlos Restrepo, Gloria Elena Suarez, Angela María Trujillo, Natalia Valencia, Hernán Darío Vásquez. **Montería:** Fabio Javier Molina, Soraya Montaño, Judi Rojas, Diana Valencia. **Neiva:** José Alexander Cabezas Gutiérrez, Nelson Ramírez Plazas. **Pasto:** María del Rosario Álvarez.
